# Efficacy and selectivity of tumor-treating field therapy for triple-negative breast cancer cells via in-house delivery device

**DOI:** 10.1007/s12672-023-00647-w

**Published:** 2023-03-29

**Authors:** Austin R. Smothers, Jason R. Henderson, John J. O’Connell, Jonathan M. Stenbeck, Delphine Dean, Tyler G. Harvey, Brian W. Booth

**Affiliations:** 1 Center for Innovative Medical Devices and Sensors (REDDI Lab), Clemson University, Clemson, SC USA; 2 Department of Bioengineering, Clemson University, Clemson, SC USA; 3 Quiverent LLC, Greenville, SC USA; 4 Prisma Health Cancer Institute, Prisma Health, Greenville, SC USA; 5 Clemson University School of Health Research, Clemson, SC USA; 6 University of South Carolina School of Medicine-Greenville, Greenville, SC USA

**Keywords:** Breast cancer, Electric field intensity, Oscillating electric fields, Radiotherapy, Triple-negative breast cancer (TNBC), Tumor-treating fields (TTFields)

## Abstract

**Purpose:**

Triple-negative breast cancer continues to be one of the leading causes of death in women, making up 7% of all cancer deaths. Tumor-treating electric fields are low-energy, low-frequency oscillating electric fields that induce an anti-proliferative effect on mitotic cells in glioblastoma multiforme, non-small cell lung cancer, and ovarian cancer. Little is known about effects of tumor-treating fields on triple-negative breast cancer and known research for tumor-treating fields only utilizes low (< 3 V/cm) electric field intensities.

**Methods:**

We have developed an in-house field delivery device capable of high levels of customization to explore a much wider variety of electric field and treatment parameters. Furthermore, we investigated the selectivity of tumor-treating field treatment between triple-negative breast cancer and human breast epithelial cells.

**Results:**

Tumor-treating fields show greatest efficacy against triple-negative breast cancer cell lines between 1 and 3 V/cm electric field intensities while having little effect on epithelial cells.

**Conclusion:**

These results provide a clear therapeutic window for tumor-treating field delivery to triple-negative breast cancer.

## Introduction

Breast cancer is the most common cancer in American women, and 1 in 8 women in the United States will develop breast cancer in her lifetime [1–4]. Triple-negative breast cancer (TNBC) makes up ~ 10–20% of breast cancers in women and is the most aggressive form of breast cancer [5–7]. Furthermore, TNBC is highly resistant to hormone therapy due to its lack of three characteristic hormone receptors: estrogen, progesterone, and human epidermal growth factor receptor 2 (HER2).

TNBC is commonly treated with combination therapy; first, tumor deposits are surgically removed, then the area is exposed to high-energy external beam radiation [7, 8]. Non-selective radiation effectively eliminates large deposits of cancer cells, but surrounding healthy tissue is also damaged. This introduces many negative side effects including sunburn-like rashes, hair loss, headaches, nausea, vomiting, and extreme fatigue [9, 10]. A more selective radiotherapy treatment method for TNBC should be explored to minimize negative side effects, improve patient quality of life, and maintain treatment effectiveness.

Oscillating electric field (OEF) treatment has been shown to be effective against brain, lung, and ovarian tumor formation both in vitro and in vivo [11–15]. During mitosis, nuclear components of cells such as microtubules, spindle fibers, and chromosomes can be influenced by OEF because they are polar (i.e., have a separation of electrical charge). These components become misaligned during the metaphase-anaphase transition as a result of OEF treatment, causing cellular stress. This leads to mitotic exit or early apoptosis, which decreases cellular proliferation [16, 17]. In clinical trials for patients with glioblastoma multiforme (GBM), median progression-free survival increased by 40% when OEF treatment was implemented as a conjunctive therapy to *temozolomide* [18] as compared to chemotherapy treatment alone [11, 13].

Tumor-treating fields (TTFields) represent a very narrow range of OEF [12]. TTField efficacy against different cell lines shows dependence on electric field intensity. Specifically, field intensities of ~ 3 V/cm show greatest treatment efficacy against TNBC cells [13]. However, treatment efficacies of field intensities beyond 3 V/cm were not investigated, and the level of selectivity of TTField treatment for TNBC versus non-cancer cells is unknown. To address these gaps, we have designed an in-house delivery device to investigate TTField therapy against TNBC in vitro over a much broader range of electric field intensities and frequencies than other known manufactured devices [11, 13, 19, 20]. After validating that our device is capable of delivering TTFields at the expected output voltages, we investigated TTField efficacy against TNBC at 1.5, 3, 3.75, 4.4, and 6 V/cm, and determined the capability of this treatment method to destroy TNBC cells in vitro with minimal effects against epithelial cells.

## Materials and methods

### Design and setup for TTField delivery in vitro

We have designed, built, and tested our own device capable of delivering TTFields over a broad range of electric field intensities. Our device also allows us to test an unlimited number of combinations of alternating current waveforms rather than being restricted to a single frequency or a constant peak voltage.

**Fig. 1:**
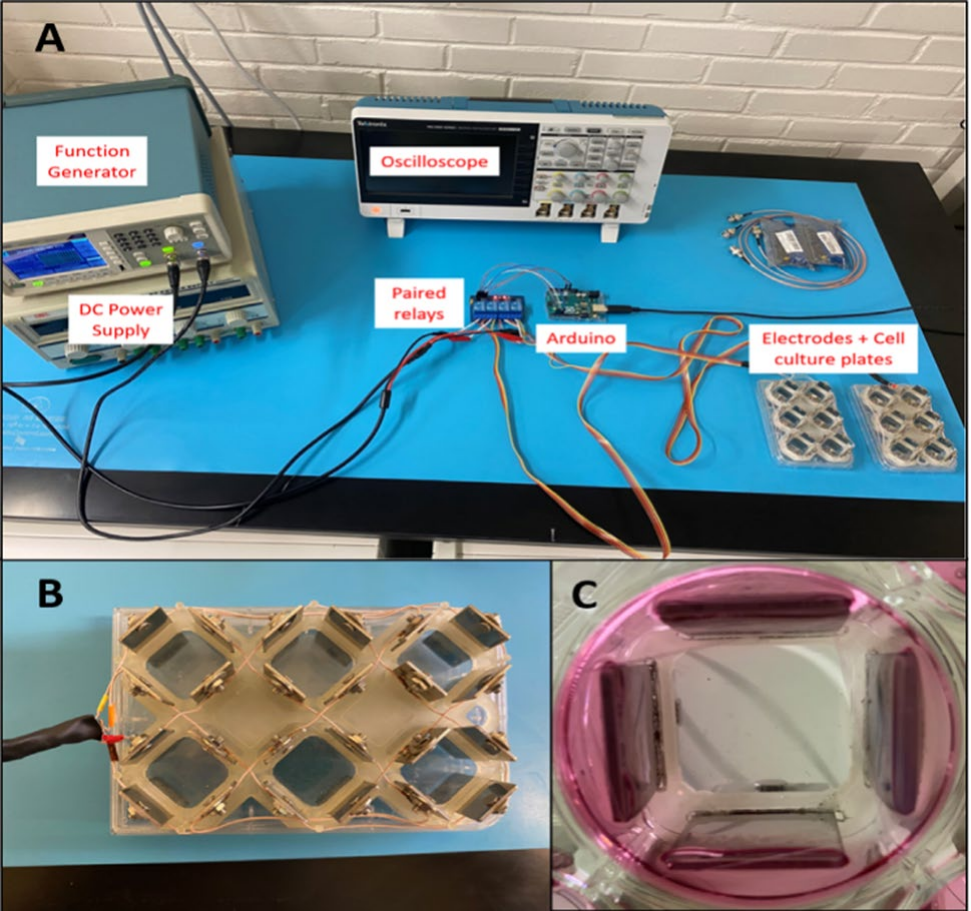
Setup and layout of TTField delivery in vitro. **A** Separate function generator channels are connected to paired relays that control the activation of parallel electrodes. An Arduino controls commutation time and activation time of parallel electrodes. **B** Electrodes are wired together in series and fit into the lid of a 6-well cell culture plate via laser-cut slits reinforced by 3D-printed, high-temperature resin that can withstand an autoclave environment. **C** Electrodes sit within each well < 1 mm from the bottom of the well and ~ 10 mm within the cell culture media. A polystyrene microscope cover slip with cells seeded onto it sits between the electrodes

**Fig. 2:**
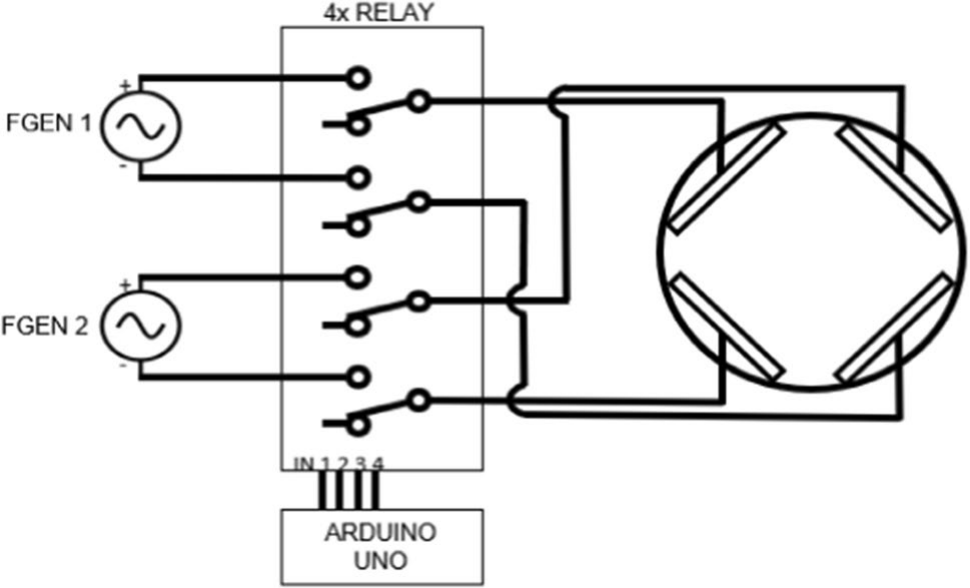
Circuit schematic of experimental setup. Each electrode can be independently disconnected from voltage and ground to isolate the function generator channels and ensure a uniform electric field parallel to the active electrodes. Electrodes in each well are wired in series so that all wells are active simultaneously

A function generator (Tektronix AFG1062, Tektronix, Inc., Beaverton, OR) is connected to a 4-channel relay module to create the desired waveforms to be delivered to cells within a 6-well cell culture plate (Figs. 1, 2). An Arduino control board (Arduino Uno Rev3 SMD, Arduino, New York, NY) is also connected to the 4-channel relay module to control multiple simultaneously alternating waveforms, their commutation times between parallel plate pairs, and the time delay between commutations. Finally, the relay module is connected to paired stainless steel electrodes fitted into a 6-well cell culture plate lid. Each electrode fits within slots that were laser-etched into the cell culture plate lid. Furthermore, the electrodes are held in place by 3D-printed high-temperature resin channels (Form 2 3D Drucker, Formlabs, Somerville, MA). Electrodes are wired as shown in Fig. 2, and waveforms were monitored using a Tektronix TBS 2000 Series Digital Oscilloscope (Tektronix, Inc., Beaverton, OR).

### Electric field models and simulations

To assess the uniformity of electric fields both within and between cell culture wells in the device, electric field modeling was performed using the AC/DC Module of COMSOL Multiphysics^Ⓡ^ version 5.4 (COMSOL, Burlington, MA). A 2-dimensional geometry representing the cross-sectional plane of the device directly above the cell culture surface was created with the electrical properties of each material assigned as in Table 1.

**Table 1:** Electrical properties of modeled geometry

Domain	Material	Relative permittivity
1	Cell culture medium	80
2	Polystyrene	2.6
3	Stainless steel	1.1 × 10^6^
4	Air	1.0006

A 7.5 V, 150 kHz sinusoidal voltage was applied to the electrodes in each well which are connected to the positive electrode of function generator channel 1 in the TTField delivery device (Fig. 2). A ground condition was applied to the opposite electrodes, and no boundary conditions were applied to adjacent electrodes (though they still retained the inherent electrical properties of stainless steel.)

**Fig. 3:**
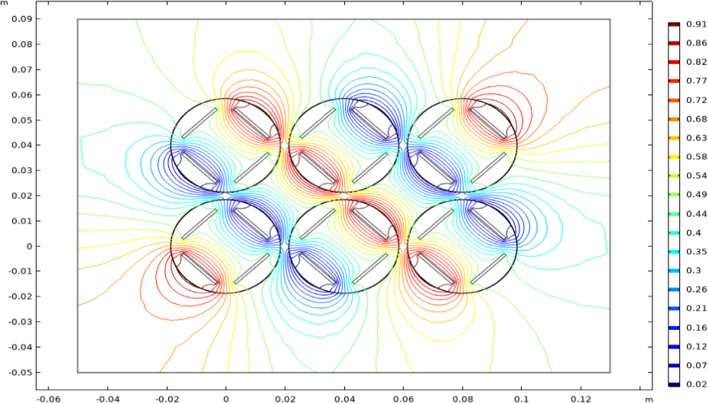
2D contour plot of simulated electric potential. The model showed a uniformity of the electric field between each well. A region of roughly uniform field shape was identified in the center of each well as a site for cell culture. This uniformity also shows that electric fields within each well do not affect those in other wells

Free-triangular meshing was performed with physics-controlled settings at a “Normal” level (135,664 triangles, minimum element quality 0.4776, average element quality 0.8331) and a time-dependent study was computed for a range of times from 0 to 6.67 μs (representing one full period of the sinusoidal signal) with an increment of 0.0667 μs, using a Direct Time Dependent (MUMPS) solver configuration with a relative tolerance of 0.01, memory allocation factor of 1.2, and pivot threshold of 0.1. A 2D contour plot of the resulting electric potential was generated for each time point. A representative image depicting time 0.267 μs is shown in Fig. 3.

### Sterilization, cleaning, and other potential issues

Issues regarding contamination and temperature control must be accounted for due to the direct contact between the electrodes and cell culture media (Fig. 1). Prior to each treatment, the electrodes are soaked in 70% ethanol for 15 min, then allowed to air-dry for 10 min. Using this decontamination method, we placed the electrodes in plain cell culture media for over 72 h and observed no microbial contamination or deterioration to the electrodes.

A thermistor was used to monitor device and setup temperature, while a thermometer was used to monitor cell culture media temperature to ensure that heat dissipation had no adverse effect on cell growth. Furthermore, there were no signs of electrophoresis or pH change when the electrodes were placed in empty cell culture media for 72 h while in use at electric field intensities below 4 V/cm. Importantly, this is not true for field intensities at or above 4 V/cm which can affect natural cell growth.

Due to some cells attaching and growing on well surfaces outside of the electrodes, plastic microscope coverslips were used to isolate cells between the electrodes. Furthermore, it was determined via field modeling that the region of uniform electric fields within each well is approximately the dimensions of a cover slip in the center of each well. Cells were seeded onto the coverslip and allowed to adhere. After treatment, the coverslips were transferred to a new, empty six-well cell culture plate for the most accurate cell counts. Otherwise, untreated or non-uniformly treated cells residing outside of the treatment area would be included in these counts.

### Electric field parameters and delivery

To ensure that our setup is able to adequately deliver TTFields to cells within culture plate wells, we designed a protocol combining the treatment parameters of Kirson et al. [14] and Berkelmann et al*.* [19]; TTFields were delivered at a frequency of 150 kHz with a 1-min commutation time (as opposed to 0.25–1 s which all others using Inovitro™ are limited to). For device validation, an electric field intensity of 1.5 V/cm was used. Further investigation used 1.5, 3, 3.75, 4.4, and 6.0 V/cm electric field intensities following device validation. Non-stop treatment was delivered to cells for 24, 48, and 72 h. After each time point, viable cell counts via flow cytometry were recorded (*n* = 5) and microscope images were taken following each treatment.

### Cell culture parameters

MDA-MB-231 and HCC38 (human TNBC) cell cultures were grown in DMEM plus 10% fetal bovine serum (FBS) and 1% antibiotic–antimycotic (anti-anti) in a CO_2_ incubator (5% CO_2_) at 37 °C. MCF-12 (human breast epithelial) cell cultures were grown in DMEM with MEGM Growth Supplements Singlequots™, plus 10% FBS and 1% anti-anti at the same incubator conditions. 484 mm^2^ plastic microscope coverslips were sterilized using 70% ethanol and placed into each well of a 6-well cell culture plate (Corning Inc., Corning, NY). Cells were seeded at ∼100,000 cells per well onto each coverslip, and additional cell culture media was added to achieve a 2 mL total volume solution per well. Cells were given 24–48 h after seeding to adhere before starting treatment. Treatment was then delivered to cells while being grown in the incubator.

### Microscopy and flow cytometry

Brightfield microscope images were taken using a Zeiss^®^ Axiovert 40 CFL inverted fluorescence phase contrast microscope and Axiovision Rel. 4.8 imaging software (Carl Zeiss Industrial Metrology, Maple Grove, MN). An ORFLO^®^ Moxi Go II flow cytometer (ORFLO Technologies, Ketchum, ID) with single-channel cassettes was used to perform cell size and viability counts. When taking viable cell counts using flow cytometry, data for each cell line were gated based on the measured mean diameter of the cell ± three standard deviations of the mean based on a sample size of *n* = 5 from experimentation during device validation. Diameters for MDA-MB-231 and HCC38 cells were further validated by comparison to previous studies [22, 23], but reliable previous data for MCF-12 cells could not be found.

### Scratch test

MDA-MB-231 and MCF-12 cells were seeded at 100,000 cells per well and allowed to grow to confluence (~ 72 h after initial seeding). A 200 μL micropipette tip was used to create a “wound” within each culture (*n* = 3), and the initial diameter of the wound was measured and recorded in three different locations using EVOS FL Auto PearlScope64 imaging software (Life Technologies, Carlsbad, CA), and then averaged. TTField treatment was given to the cells for 24 h, and the diameter of the wound was measured, recorded, and averaged once more. Data is presented as percent change in diameter.

### Data interpretation and statistical analysis

For all quantitative data, a standard one-way ANOVA followed by a *t*-test was performed to determine statistical significance. For investigating the relationship between treatment efficacy and electric field intensity, statistical significance for both TNBC cell lines was determined in relation to epithelial cells at the same field intensity rather than the untreated group of the corresponding line. There was no blindedness involved with experimentation.

## Results

### Electric field models and simulations

The electric potential contour plot (Fig. 3) at each time point was assessed visually for uniformity between wells of the cell culture plate and to identify regions of a roughly uniform electric field within each well in which to culture cells.

### Device validation and performance at 1.5 V/cm electric field intensity

**Fig. 4:**
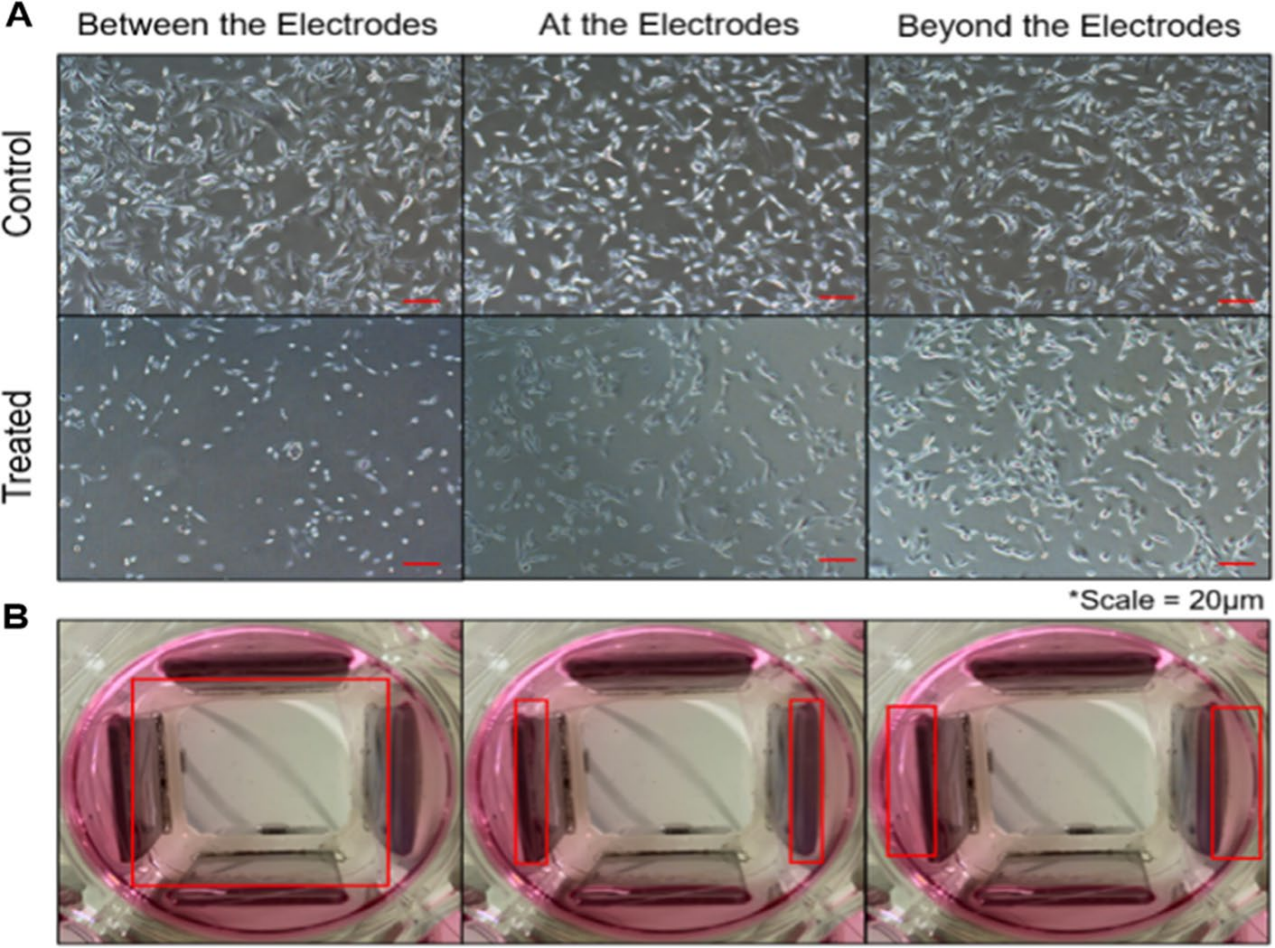
Cell numbers reduced within field following 24 h of TTField treatment. **A, B** Microscope images of treated cells show that cells within the treatment area (between the electrodes) have much lower confluence than those outside of the treatment area (beyond the electrodes)

**Fig. 5:**
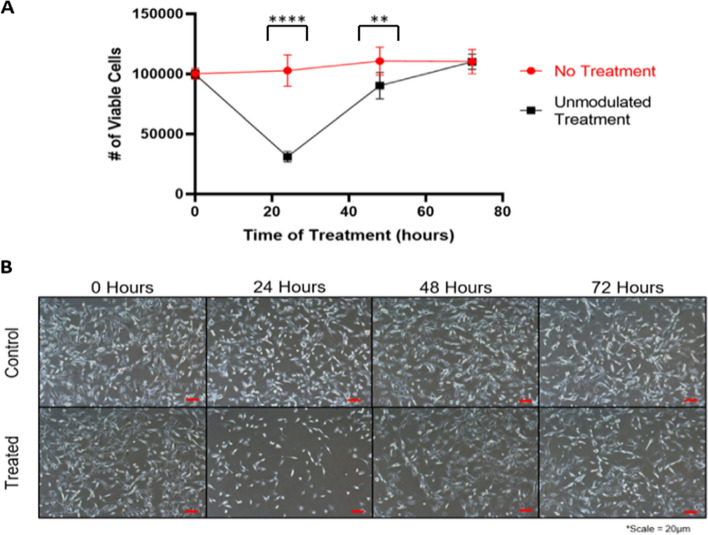
Effects of TTFields on TNBC cells after 24, 48, and 72 h of TTField treatment at 1.5 V/cm. **A, B** TNBC cell numbers drop significantly after 24 h of treatment before rebounding to control cell counts at 72 h. (*P* < 0.05 = *, *P* < 0.01 = **, *P* < 0.001 = ***, *P* < 0.0001 = ****)

After 24 h of uninterrupted exposure to uniform TTFields, TNBC cells growing within the electrodes had a stunted proliferative rate relative to those growing outside of the electrodes (Fig. 4). This was observed both quantitatively (*n* = 5) and qualitatively via flow cytometry and microscope images, respectively. Cells experienced a drastic decrease in count (*P* < 0.0001) following 24 h of TTField treatment, dropping to just 30% of confluence relative to control (Fig. 5). However, treated cells began to recover quickly after 48 h, reaching 74% of control confluence (*P* = 0.0015). Finally, after 72 h, treated cells had completely recovered and reached maximum confluence (*P* > 0.9999) (Fig. 5).

**Fig. 6:**
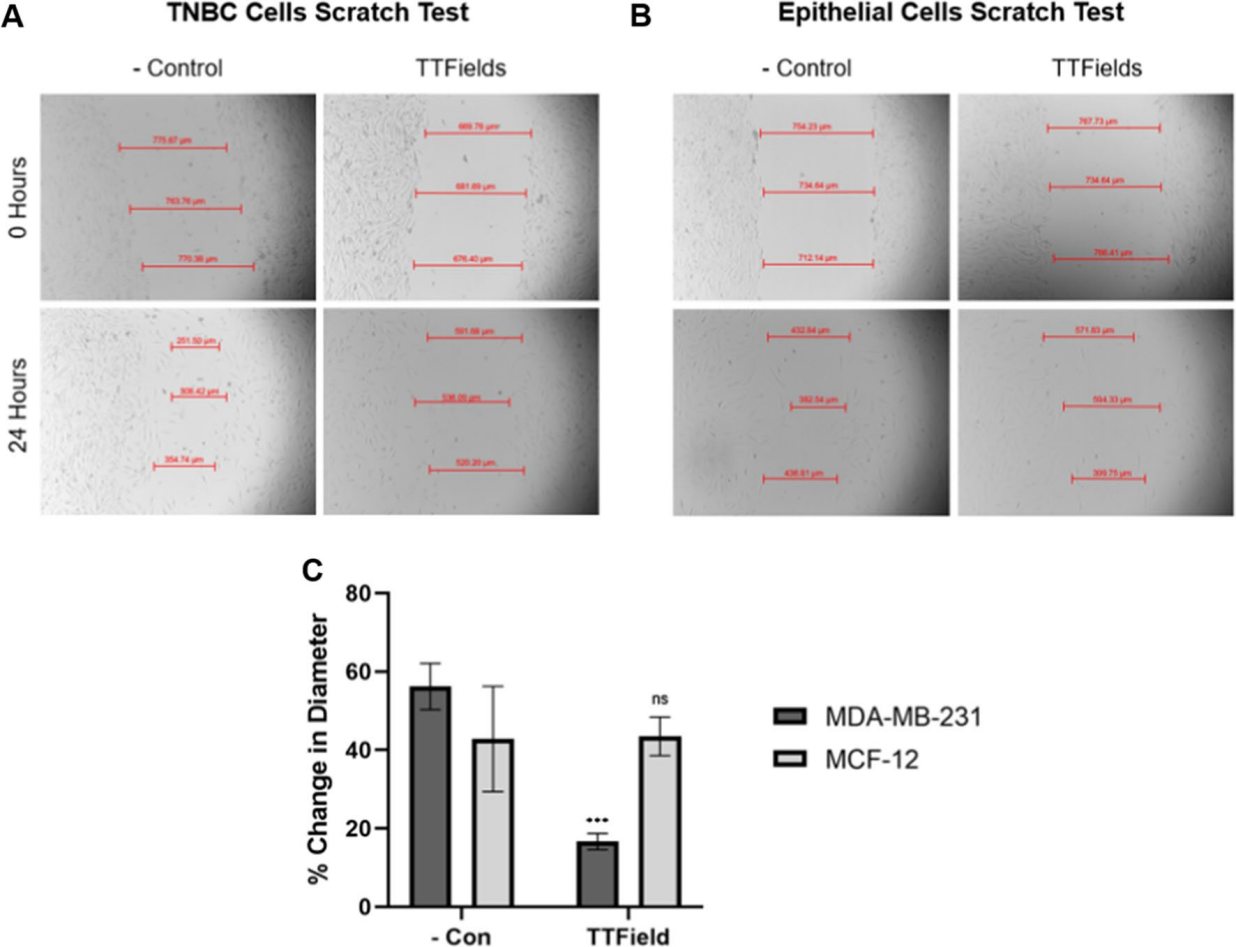
A scratch test for **A** TNBC and **B** epithelial cells after 24 h. TNBC cells were much less likely to occupy the affected area than epithelial cells after receiving 24 h of TTField treatment; the diameter of the wound in TNBC cells stayed much more consistent throughout its entirety as compared to that of epithelial cells. **C** Measurements were performed on an additional two replicates to the ones shown for a sample size of n = 3. (P < 0.05 = *, P < 0.01 = **, P < 0.001 = ***, P < 0.0001 = ****)

A scratch test was performed for both TNBC and epithelial cells to further investigate anti-proliferative effects of TTFields. After 24 h of receiving TTField treatment, TNBC cell wound diameter decreased by just ~ 17%, whereas untreated cultures showed a wound diameter decrease of ~ 56% (*P* = 0.0004). Conversely, epithelial cell wound diameter decreased by ~ 43% for both TTField treatment and the negative control (*P* = 0.9373) (Fig. 6). Additionally, epithelial cells were observed within the wound area more often than TNBC cells following 24-h treatment.

### Treatment efficacy by electric field intensity

**Fig. 7:**
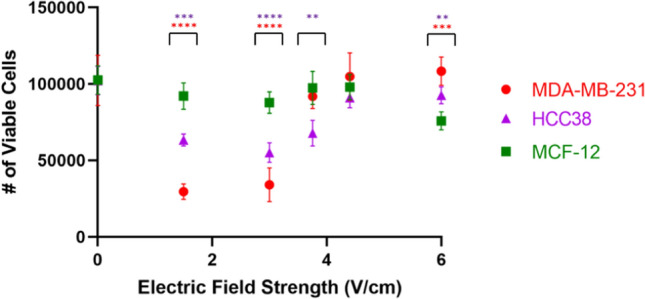
Effects of TTField intensity on treatment efficacy and selectivity for TNBC and epithelial cells. TNBC cells (MDA-MB-231, HCC38) are significantly susceptible to lower TTField intensities than breast epithelial cells (MCF-12). All statistical significance compares TNBC cells to breast epithelial cells at the same electric field intensity (*P* < 0.05 = *, *P* < 0.01 = **, *P* < 0.001 = ***, *P* < 0.0001 = ****)

We investigated anti-proliferative effects of electric fields with intensities of 1.5, 3, 3.75, 4.4, and 6 V/cm on MDA-MB-231, HCC38, and MCF-12 cells using the same frequency (150 kHz) and commutation time (1 min) as our validation study. For both TNBC cell lines, treatment efficacy was highest at 1.5 and 3.0 V/cm (*P* < 0.0001 for all points) albeit slightly less for HCC38 cells (Fig. 7). At 3.75 V/cm, treatment had no observable effects on MDA-MB-231 cells (*P* = 0.3762), but was still somewhat effective against cell proliferation in HCC38 cells (*P* = 0.0012). At 4.4 V/cm, TTFields had no observable effect on either cell lines (*P* = 0.4086 for MDA-MB-231, *P* = 0.1783 for HCC38). When approaching 6 V/cm, MCF-12 cells experienced much higher death than either MDA-MB-231 cells (*P* = 0.0002) or HCC38 cells (*P* = 0.0017).

## Discussion

Our device shows great efficacy in treatment against TNBC cells. This is especially indicated by the ability for cells to grow unharmed outside of the electrodes while cells within the electrodes had significantly slowed growth (Fig. 5). This supports that our setup not only was successful in destroying TNBC cells, but also that the cells were dying as a result of the treatment and not from other unknown factors. Furthermore, this method of treatment is selective at combating TNBC cell growth while having minimal effect against epithelial cells within 1–3 V/cm, indicating a clear therapeutic window for treatment within this range of field intensities. However, TNBC cells began to immediately recover after 24 h. We believe this is due to the cells becoming sensitized to the treatment, as is typical with other forms of radiotherapy and chemotherapy, and is a result of the plasticity of TNBC cells. This is especially common in basal-like cancer subtypes such as the MDA-MB-231 cell line [24, 25]. We hypothesize that the remaining cells after 24 h of treatment may be a quiescent cancer stem cell population that also maintain high plasticity and resistance to treatment [26].

While TTFields were effective in preventing TNBC cell growth, this effect was to varying degrees between TNBC cell lines. TTFields had greater treatment efficacy against MDA-MB-231 cells within a shorter range of electric field intensities while showing moderate efficacy against HCC38 cells over a broader range. This could be explained by differences in cell sizes. MDA-MB-231 cells were 21.55 ± 0.39 μm (*n* = 5 from control after 24 h of growth) and HCC38 cells were 26.82 ± 0.47 μm (*n* = 5 from control after 24 h of growth). This relatively large difference in diameter between cell lines would result in differences in dipole moments which can affect the magnitude of influence from the electric fields on the cells.

Our setup was very effective in treating TNBC in vitro, especially at electric field intensities between 1 and 3 V/cm (Fig. 7), but can still be improved. At field intensities above 6 V/cm, we observed a higher rate of cell death in epithelial cells than TNBC cells. This could be due to electrophoresis occurring within the cell culture media caused by the high voltage outputs of the electrodes. This would cause a pH change within the culture environment, leading to poor conditions for epithelial cell growth while having minimal effect on TNBC cells. In previous studies, MDA-MB-231 cells had maximal growth within a pH range of 6.7–7.4 [27], while human epidermal cells had maximal growth within a much narrower pH range of 7.0–7.2 [28]. This suggests that TNBC cells can survive in more diverse pH environments such as those caused by electrophoresis. Additionally, the simulated electrical interference from inactive stainless steel electrodes does not seem to perturb treatment but could be improved. This could be done by isolating the electrodes using a low-grade insulating material that is electrically transparent at the utilized frequencies. This could also help minimize the occurrence of electrophoresis by reducing the direct contact between the electrodes and cell culture media.

The high customizability of our device is largely due to two of its driving components: the function generator and an independent Arduino control board. This combination of components allows for control of numerous factors that can affect treatment efficacy such as frequency or amplitude modulation as well as their magnitudes of deviation, commutation times between parallel plate pairs, and the time delay between commutations. This high variability in field parameters can help address tumor populations that also contain high variability for cell age, size, shape, and ploidy to maximize treatment efficacy towards TNBC.

Despite many positive features of our setup, there are also some limitations. First, our device is limited to use with 6-well cell culture plates, as it was specifically designed for this layout. This size plate allowed for easier construction of the electrodes and their housing, but removed the ability to use other well sizes. Our setup is also limited by the number of electrode sets available; we can only treat up to two plates of cells at a time simultaneously. Increasing treatment throughput would require another of the entire setup shown in Fig. 1. Finally, our setup is limited to in vitro investigation and is only in two-dimensional mono-cultures. Performing this experimentation for co-cultures, three-dimensional tumorspheres, or with in vivo animal models would more accurately represent natural tumor growth and treatment.

It is not suggested to investigate electric field intensities beyond 6 V/cm, as we will likely continue to see an inverse relationship between electric field intensity and epithelial cell count while TNBC cell count would remain unaffected. Furthermore, this setup requires a robust power source that can withstand high voltage and current outputs, as 30 V was the minimum required voltage to support field intensities of at least 6 V/cm using the separation distance of our electrodes. This poses a significant practical issue for in vivo research and patient treatment due to drastically increased separation distances between electrodes, especially for battery-powered setups such as the NovoTTF-100A™ used in clinical trials.

Given the heterogeneity of TNBC cells within a cell population [12–14] and that the ideal electric field frequency and intensity for maximal cell death vary for cells in differing stages of mitosis [24], researchers should assess if treatment across a spectrum of average frequencies is more lethal than that of a single, discrete frequency. To address this, future experimentation utilizing similar methodology should include frequency modulation (FM), amplitude modulation (AM), combined frequency and amplitude modulation, or the addition of fractionated ionizing radiation (IR) therapy.

## Data Availability

All data generated or analysed during this study are included in this published article.
